# Predictors of Hospital Mortality in Patients with Acute Coronary Syndrome Complicated by Cardiogenic Shock

**DOI:** 10.3390/s21030969

**Published:** 2021-02-01

**Authors:** Gábor Tamás Szabó, András Ágoston, Gábor Csató, Ildikó Rácz, Tamás Bárány, Gábor Uzonyi, Miklós Szokol, Balázs Sármán, Éva Jebelovszki, István Ferenc Édes, Dániel Czuriga, Rudolf Kolozsvári, Zoltán Csanádi, István Édes, Zsolt Kőszegi

**Affiliations:** 1 Department of Cardiology, Faculty of Medicine, University of Debrecen, 4032 Debrecen, Hungary; iracz73@gmail.com (I.R.); drbaranytamas@gmail.com (T.B.); miklos.szokol@gmail.com (M.S.); dczuriga@med.unideb.hu (D.C.); rudlinghealth@yahoo.com (R.K.); csanadi.zoltan@med.unideb.hu (Z.C.); edes@med.unideb.hu (I.É.); koszegi@med.unideb.hu (Z.K.); 2 The III: Department of Internal Medicine, Szabolcs–Szatmár–Bereg County Hospitals and University Teaching Hospital, 4400 Nyíregyháza, Hungary; agostonandras48@gmail.com; 3 Hungarian National Ambulance Service, 1024 Budapest, Hungary; csatogabor@yahoo.com; 4 Department of Cardiology, Uzsoki Hospital, 1145 Budapest, Hungary; uzonyi.gabor@uzsoki.hu (G.U.); sarman.balazs@uzsoki.hu (B.S.); 5 Department of Cardiology, Faculty of Medicine, University of Szeged, 6725 Szeged, Hungary; jebelovszki@freemail.hu; 6 Heart and Vascular Center, Semmelweis University, 1122 Budapest, Hungary; edes.istvan@kardio.sote.hu

**Keywords:** telemedicine, prehospital triage, acute heart failure, myocardial perfusion

## Abstract

As demonstrated by earlier studies, pre-hospital triage with trans-telephonic electrocardiogram (TTECG) and direct referral for catheter therapy shows great value in the management of out-of-hospital chest pain emergencies. It does not only improve in-hospital mortality in ST-segment elevation myocardial infarction, but it has also been identified as an independent predictor of higher in-hospital survival rate. Since TTECG-facilitated triage shortens both transport time and percutaneous coronary intervention (PCI)-related procedural time intervals, it was hypothesized that even high-risk patients with acute coronary syndrome (ACS) and cardiogenic shock (CS) might also benefit from TTECG-based triage. Here, we decided to examine our database for new triage- and left ventricular (LV) function-related parameters that can influence in-hospital mortality in ACS complicated by CS. ACS patients were divided into two groups, namely, (1) hospital death patients (*n* = 77), and (2) hospital survivors (control, *n* = 210). Interestingly, TTECG-based consultation and triage of CS and ACS patients were confirmed as significant independent predictors of lower hospital mortality risk (odds ratio (OR) 0.40, confidence interval (CI) 0.21–0.76, *p* = 0.0049). Regarding LV function and blood chemistry, a good myocardial reperfusion after PCI (high area at risk (AAR) blush score/AAR LV segment number; OR 0.85, CI 0.78–0.98, *p* = 0.0178) and high glomerular filtration rate (GFR) value at the time of hospital admission (OR 0.97, CI 0.96–0.99, *p* = 0.0042) were the most crucial independent predictors of a decreased risk of in-hospital mortality in this model. At the same time, a prolonged time interval between symptom onset and hospital admission, successful resuscitation, and higher peak creatine kinase activity were the most important independent predictors for an increased risk of in-hospital mortality. In ACS patients with CS, (1) an early TTECG-based teleconsultation and triage, as well as (2) good myocardial perfusion after PCI and a high GFR value at the time of hospital admission, appear as major independent predictors of a lower in-hospital mortality rate.

## 1. Introduction

Ischaemic heart disease is one of the common causes of death worldwide and its frequency is increasing [1]. Recent guidelines [2,3] state that the timely diagnosis of acute coronary syndrome (ACS) and the elimination of any delay are the key factors of an improved outcome. In most ACS cases, the first medical contact takes place far from hospitals, usually initiated by the paramedic staff. Therefore, in the vast majority of cases, the primary, pre-hospital diagnosis of ACS is based on the typical signs and symptoms of the disease and especially on the electrocardiogram (ECG) analysis [4]. Besides the traditional 12-lead ECG, trans-telephonic ECG (TTECG) can also be applied by the paramedic specialist [2]. In previous studies, ourselves and others have shown that the utilization of the TTECG and TTECG-facilitated triage of patients significantly shortened the intervention time and improved in-hospital mortality in patients with ST-segment elevation myocardial infarction (STEMI) [5,6]. ACS, including STEMI, is one of the main precipitating factors of cardiogenic shock (CS) [7,8]. It has been estimated that 5–15% of patients with ACS experience signs and symptoms of CS [9,10]. Although the prognosis of CS has gradually improved over the past few years, the hospital mortality rate is still high (20–40%), despite advances in early revascularization and the implementation of new drugs and mechanical circulatory support devices [10]. Some factors that affect mortality in ACS patients with CS have already been established. Among others, these are advanced age, anoxic brain damage, low left-ventricular ejection fraction (LVEF), deterioration in renal function, and resuscitation [7,8,10]. A patient displaying ACS and CS should be triaged to a fast track for early revascularization, with the aim of maintaining perfusion to prevent organ dysfunction.

Since the TTECG-facilitated triage of STEMI patients has shortened both transport time and percutaneous coronary intervention (PCI) procedural time intervals, and, at the same time, improved hospital survival [6], we have decided to examine our ACS patient database for CS to look for factors that affect mortality. In particular, our aim was to find new triage- and LV function-related independent predictors that may influence in-hospital mortality in CS complicating ACS.

In order to put our hypothesis to the test, we set out to retrospectively evaluate our database of ACS patients with CS (*n* = 287) for: (1) TTECG procedures, (2) various LV functional data and (3) demographic factors, comorbidities, and cardiac risk factors. Our main objective was to examine the difference between the hospital death group and the hospital survival group with the expectation that the early TTECG-facilitated triage and improved pre-hospital therapy of patients would lead to better perfusion, LV function, and improved patient survival.

## 2. Materials and Methods

### 2.1. Data Collection

**Figure 1:**
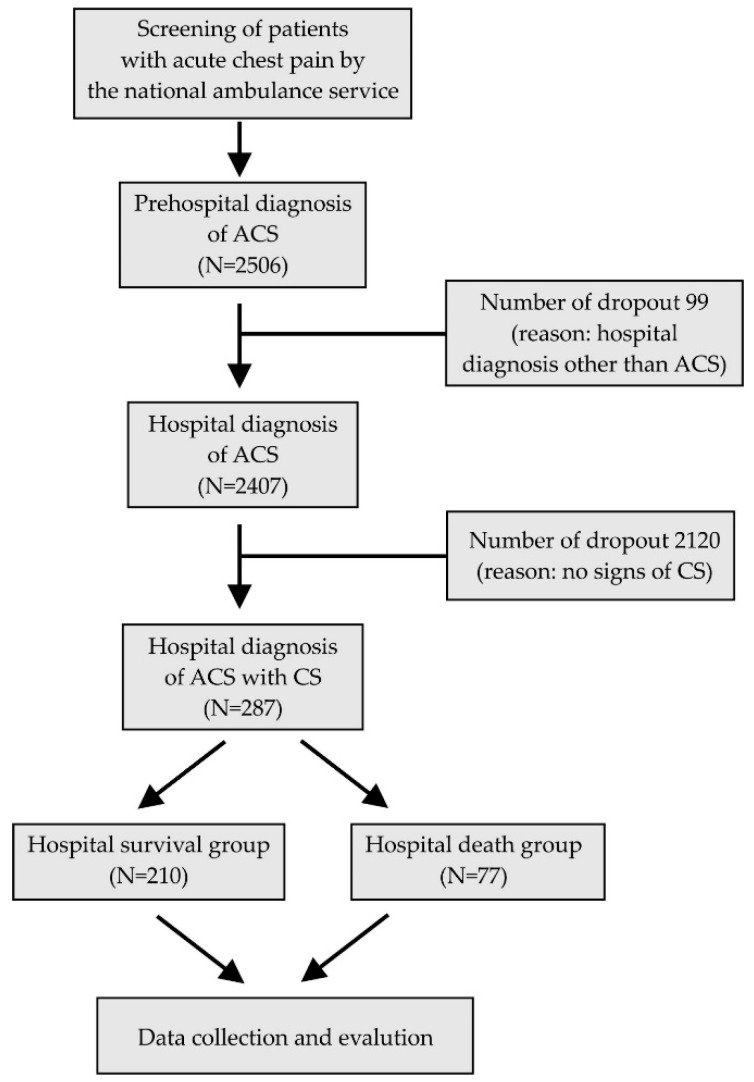
CONSORT diagram showing patient flow at each stage of the data collection. ACS = acute coronary syndrome; CS = cardiogenic shock.

The study was conducted between 1st January 2009 and 31st December 2012 at the Northern Great Plain region of Hungary as a collaborative effort between the Department of Cardiology, University of Debrecen, Hungary and the Hungarian National Ambulance Service. During this time, 2506 patients were transferred to the regional PCI centre (Department of Cardiology, University of Debrecen, Debrecen, Hungary) with a diagnosis of ACS. The patient flow is depicted in Figure 1.

Upon arrival at the PCI centre, each patient was promptly interviewed and examined by a cardiologist and the diagnosis of ACS was established. At the time of admission, laboratory blood tests were run via a series of creatine kinase (CK) measurements, renal and liver functions, as well as blood count. Based on serial CK measurements, the peak CK (CK_max_) value was determined. Following bedside echocardiography, patients were transferred to the catheterization laboratory for coronary angiography, and, when necessary, for PCI.

Among ACS patients, altogether 287 CS cases were registered (Killip class IV) [11]. The diagnosis of CS was based on hypotension (systolic blood pressure < 90 mmHg) with signs of hypoperfusion. For all ACS patients with CS, immediate echocardiography, coronary angiography, and, if possible, coronary revascularization were carried out. After fluid challenge, all ACS patients with CS received vasopressor therapy and an inotropic agent, when necessary. In the case of respiratory failure (hypoxemia or hypercapnia), the patients were intubated and ventilated. Since the study was carried out between 2009 and 2012, most of the patients (282 patients) also received device therapy in the form of intra-aortic balloon pump (IABP) support. Recently, it has been shown in the IABP-SHOCK II trial that the use of an IABP did not actually improve outcomes in patients suffering from ACS and CS [12,13]. Therefore, in recent guidelines the routine use of an IABP is not recommended [10,14].

ACS patients with CS were assigned to the following two groups: (1) hospital death patients (*n* = 77) and (2) hospital survivors (control, *n* = 210). The primary aim of this study was to examine the efficacy outcomes of triage- and LV function-related parameters affecting in-hospital mortality.

The TTECG methodology was previously described in detail [6]. TTECG was performed with a portable, 12-lead, battery-operated system (HeartView P12/8 Plus by Aerotel). The device was supplemented with 3 external, cable-connected electrodes and 4 embedded electrodes on the back of the main unit. This layout allowed the recording of both limb and precordial leads. A 2.5 s interval of each lead and a 10 s interval of the rhythm strip (lead II) were recorded with a sampling rate of 375 samples/s, producing a standard 12-lead ECG layout. The radiotelephone system of the Hungarian National Ambulance Service (Tetra) was used for data transmission.

Paramedic specialists participating in the trial were trained for emergency cardiac service and advanced cardiovascular life support. Before the study, the specialists were instructed how to evaluate patients with chest pain (with a presumptive diagnosis of ACS) at the scene and administer acetylsalicylic acid, sodium heparin, narcotics, and other medications if necessary. All units were uniformly equipped with both conventional ECG and portable TTECG systems. The conventional ECG machine recorded 12 leads in 4 consecutive steps at a standard paper speed (25 mm/s). Conventional ECG was always recorded by the emergency team at the first contact. However, during the period of the study, it was not obligatory, just an option for the paramedic team to record and transmit a TTECG to the PCI centre. Some teams obtained and transferred TTECGs from all ACS patients, while other teams did so only if they encountered problems with the clinical diagnosis, medical treatment, and the interpretation of the ECG. Typically, when the patient was located farther away from the PCI centre (rural areas), TTECG-based triage was used more frequently by emergency teams. Once the TTECG signal was transmitted to the PCI centre, the patient’s relevant clinical data were discussed via teleconsultation.

Coronary angiography investigations were performed with the help of a Philips Integris CV device, cine loops were recorded at 15 frames/s using 300 mg iodine/mL non-ionic contrast material. The primary PCI procedure was performed in a standard way. The left ventricular (LV) area at risk (AAR) calculation was based on the analysis of coronary angiograms from multiple projections with a validated computer software (Holistic Coronary Care; HCC) [15]. To represent most of the individual variations of the coronary circulation, this program uses a modified coronary classification method, resulting in 12 different coronary artery circulation types.

As the blood supply of the myocardium can be more adequately quantified by evaluating myocardial perfusion than visualizing the epicardial flow on the angiogram [16], myocardial reperfusion after PCI was assessed by the myocardial blush grade obtained using a computer program (Quantitative Blush Evaluator; QuBE) [17,18]. The blush grade results (QuBE score) were expressed in arbitrary units for the whole LV (LV QuBE score) or for segments in the AAR (total AAR QuBE score and AAR QuBE score/AAR segments number).

All patients underwent an echocardiographic examination before and after the intervention, using an Acuson–Sequoia device with a 3.5 MHz harmonic imaging transducer. LV ejection fraction (LVEF) calculation was performed using the Simpson’s formula of the software package.

The time interval between symptom onset and hospital admission was the duration of time between the onset of typical chest pain and the arrival of the ambulance service unit at the PCI centre. Door-to-balloon time was defined as the time interval between the arrival of the ambulance service unit at the PCI centre and the inflation of the intracoronary balloon in the catheterization laboratory.

The study was conducted in accordance with the Declaration of Helsinki. Study data were collected after receiving the written consent of the patients or their relatives. Data management and collection procedures were approved by the institutional review boards of the Department of Cardiology, University of Debrecen, Hungary and the Hungarian National Ambulance Service.

### 2.2. Statistical Analysis

A statistical analysis was performed by the GB-STAT v8.0 program. Depending on the type of variable in question, various statistical approaches were applied: calculation of the mean and standard deviations (SD) or calculation of the absolute and relative frequencies. Normally distributed continuous variables were compared by using Student’s t test at an α-level of 5%; while comparative analysis of categorical variables was carried out using nonparametric statistics (Wilcoxon–Mann–Whitney test) at a α-level of 5%.

All predetermined variables between the hospital death group and the hospital survival group were assessed by applying a univariate logistic regression model. Odds ratios and 95% confidence intervals (CI) were calculated. Variables characterized by a *p* < 0.20 in the univariate analysis were selected for multiple regression and were quantified for adjusted odds ratios and CIs for in-hospital mortality. A *p* value of <0.05 was regarded as significant. For cumulative survival analysis, the Cox regression model was used.

## 3. Results

**Figure 2:**
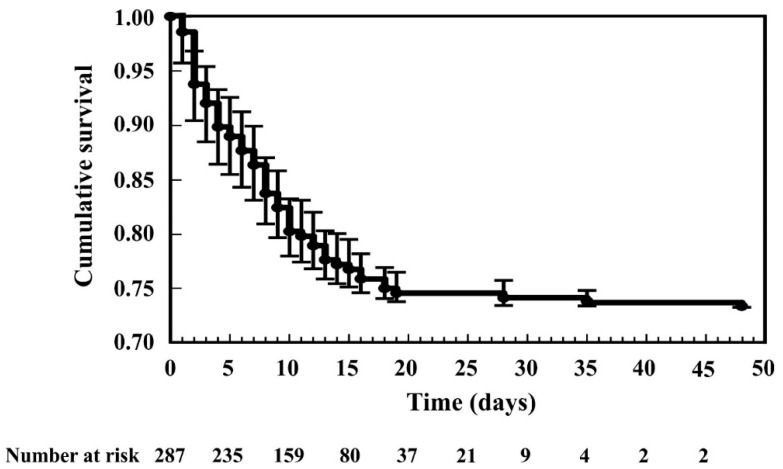
The Kaplan–Meier curve depicts the in-hospital survival rate (value ± 95% confidence intervals) of all the acute coronary syndrome patients with cardiogenic shock in the first 48 days.

Altogether, 287 ACS patients with CS were involved in the study (77 patients in the hospital death group and 210 patients in the hospital survival group). The total in-hospital mortality was 26.83% (77 patients). The cause of death was progression of CS, asystole, cardiac tamponade, arrhythmias, stent thrombosis, and infection. The frequency of death was highest in the first week of hospitalization (47 deaths occurred in the first week). After day 48, no hospital death occurred in this patient’s cohort (Figure 2).

The one-year mortality of ACS patients with CS was 33.45% (96 patients). The mean time spent in hospital was 11.45 ± 7.86 days, with an average duration of IABP treatment of 4.82 ± 3.82 days. The number of IABP-related vascular complications was 16 cases (pseudoaneurysm, leg ischaemia, thromboembolic complications, or severe haematoma).

Table 1 shows the baseline characteristics of the hospital death and survival groups.

The two groups did not appear to be significantly different with regard to risk factors and earlier medical history. Still, patients in the hospital death group were moderately, but not significantly (*p* = 0.1425) older than the hospital survivors. An examination of the blood chemistry of patients revealed significantly higher CK_max_ and a lower baseline glomerular filtration rate (GFR) values in the hospital death group compared to the survivor group (*p* = 0.0038 and *p* < 0.0001, respectively). Significantly more (*p* = 0.0104) TTECG-based consultations were performed in the hospital survival group than in the hospital death group. As expected, the rates of successful pre-hospital and hospital resuscitations were slightly higher in the hospital death group in comparison with the survivor group, nevertheless, the difference did not prove to be statistically significant. The examination of time intervals between symptom onset and hospital admission (Table 1) showed significant variations; however, this parameter was significantly longer (*p* < 0.0001) in the hospital death group compared to survivors. The selective analysis of hospital death group data revealed that TTECG was associated with significantly (*p* < 0.0065) shortened time intervals (in hours) between symptom onset and hospital admission (mean ± SD: hospital death with TTECG 6.75 ± 6.77 h, *n* = 21; hospital death without TTECG 34.98 ± 62.49 h, *n* = 56; all hospital death 26.63 ± 54.93 h, *n* = 77). However, this phenomenon was substantially mitigated in this group due to the low number of patients with TTECG.

The effect of TTECG-based triage on the time interval between symptom onset and hospital admission was also directly examined. For this purpose, we created two groups: TTECG group (*n* = 116) and control group without TTECG (*n* = 171). The following results (mean ± SD, in hours) were noted: TTECG group, 9.44 ± 11.21; control group, 23.77 ± 48.63 (*p* < 0.0001). Based on these data, it is obvious that TTECG-mediated triage was associated with a drastically shortened time interval between symptom onset and hospital admission.

Stent procedural data and the medical therapy introduced in the acute phase of the disease are summarized in Table 2.

In the case of 214 patients, the clinical diagnosis was CS and STEMI. The remaining cases (73 patients) were classified as CS and non-ST-elevation myocardial infarction (NSTEMI). Stents were deployed in 76.31% of the patients (hospital death group: 83.12%, survivor group: 73.81%). The door-to-balloon time of PCI patients was slightly but not significantly (*p* = 0.1369) longer in the hospital death group, compared to hospital survivors. Since the study was carried out between 2009 and 2012, a relatively low percent of drug-eluting stents was used (hospital death group: 5.19%, survivor group: 10.00%). In the case of 68 patients, no stent implantation was carried out. The reasons for not implanting stents were the following: 48 patients were referred for immediate cardiac surgery (three-vessel coronary artery disease and mechanical complications), 17 patients received balloon- or drug-eluting balloon angioplasty, 13 patients had multivessel disease with chronic total occlusions (not suitable for revascularization) and in 10 patients the stent implantation was technically not successful. Some patients had more than one reason for not having stent implantation. Considering stent procedural details, no significant difference was found between the two groups, as demonstrated in Table 2.

All patients received vasopressors and/or inotropes (there was no significant difference between the two groups). Moreover, no significant difference was observed when comparing the hospital death group with the survival group from the aspect of levosimendan and glycoprotein (GP) IIb/IIIa inhibitor (eptifibatide) administration during the acute phase of the disease (first week).

Examining the coronary angiograms of patients using the HCC program revealed that the AAR of the LV was slightly, but not significantly larger in the hospital death group than in the survivor group (*p* = 0.1571, Table 1). The above finding was in line with the significantly higher CK_max_ and lower LVEF values found by echocardiography in the hospital death group.

In the hospital survivor group, significantly higher QuBE scores values (LV QuBE score, total AAR QuBE score and AAR QuBE score/AAR segments number) were noted following PCI (Table 1) than in the hospital death group. The significant differences in QuBE scores between the two groups suggest better myocardial reperfusion and microvascular function in the survivor group.

**Figure 3:**
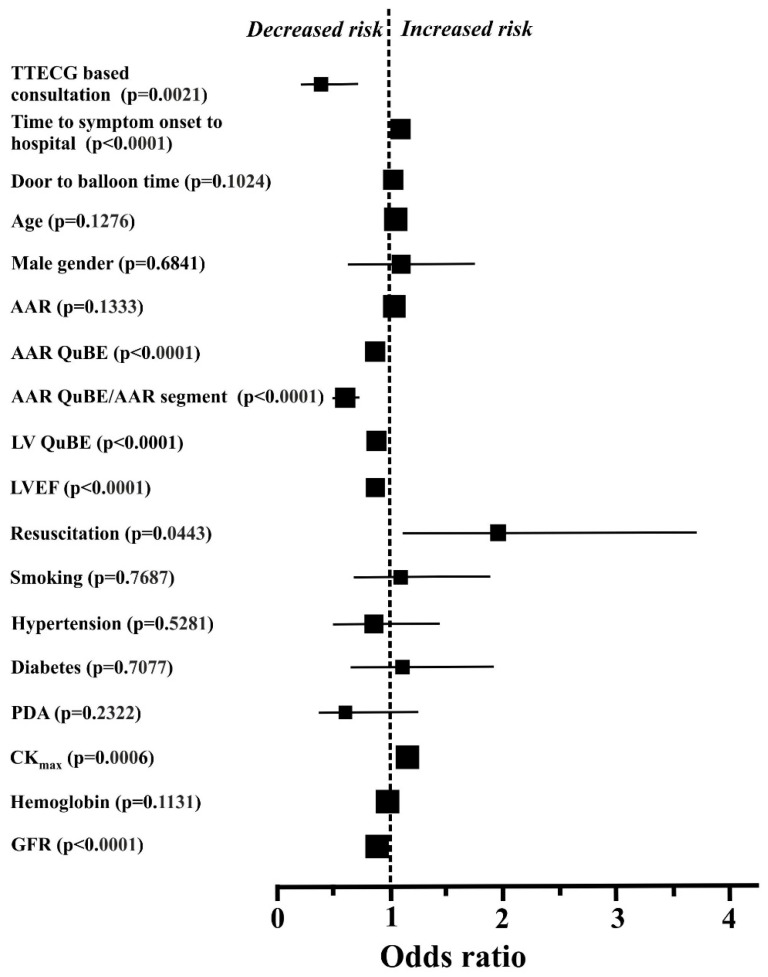
Odds ratios and 95% confidence intervals for hospital mortality in the individual subgroups, defined on the basis of baseline characteristics, blood chemistry, and percutaneous coronary intervention-related procedural data. Only selected parameters, and variables displaying a *p* value of *p* < 0.2 with the comparative analysis are shown. The sizes of the symbols reflect the number of patients in each group. For some parameters (time to symptom onset to hospital, door to balloon time, age, AAR, AAR QuBE, LV QuBE, LVEF, CK_max_, hemoglobin, and GFR), confidence intervals are within the symbols. AAR = area at risk; CK_max_ = peak creatine kinase level; GFR = glomerular filtration rate; LV = left ventricle; LVEF = left ventricular ejection fraction; PAD = peripheral artery disease; QuBE = blush score; TTECG = transtelephonic electrocardiogram.

All the predetermined parameters between the two groups were evaluated by the univariate log-rank test (Figure 3), also, odds ratios and CIs for in-hospital mortality rate were calculated. Applying the univariate statistical method, several blood chemistry parameters, LV functional data, time interval between symptom onset and hospital admission, resuscitation, and the TTECG-based consultation turned out to exert significant effect on the in-hospital mortality rate of ACS patients with CS.

Our data analysis showed that prolonged time interval between symptom onset and hospital admission, successful resuscitation and higher CK_max_ activity were linked to significantly increased odds ratios for mortality (Figure 3). Additionally, higher LVEF values, good reperfusion after PCI (high QuBE scores), and TTECG-based triage significantly lowered the odds ratios of in-hospital mortality (Figure 3).

Variables characterized by a *p* < 0.20 in the univariate analysis were selected for multiple regression and were quantified for adjusted odds ratios and CIs for in-hospital mortality. Interestingly enough, the TTECG-based consultation and triage were confirmed as significant independent predictors of lower risk (odds ratio 0.40, CI 0.21–0.76, *p* = 0.0049). Regarding LV function and blood chemistry, a high AAR QuBE score/AAR segment number ratio (odds ratio 0.85, CI 0.78–0.98, *p* = 0.0178) and higher GRF values (odds ratio 0.97, CI 0.96–0.99, *p* = 0.0042) at the time of admission were the most important independent predictors of decreased in-hospital mortality rate in this model. At the same time, prolonged time interval between symptom onset and hospital admission (odds ratio 1.010, CI 1.004–1.014, *p* = 0.0006), successful resuscitation (odds ratio 1.58, CI 1.01–3.10, *p* = 0.0411) and high CK_max_ values (odds ratio 1.16, CI 1.04–1.30, *p* = 0.0084) were the major independent predictors of an increased risk of in-hospital mortality rate.

All other parameters which were below *p* < 0.20 in the univariate analysis (i.e., LV QuBE score, AAR QuBE score, door-to-balloon time, LVEF, age, and AAR) did not prove to be significant independent predictors of the in-hospital mortality rate in our model.

## 4. Discussion

In previous studies, TTECG-based consultation with cardiologists contributed to bringing about a significantly lower in-hospital mortality rate of STEMI patients [5,6]. Moreover, TTECG-based triage has been identified as an independent predictor of decreased hospital mortality [19]. This result was somewhat unexpected, and it was presumed that the benefits related to TTECG-based consultation were associated with more favourable pre-hospital medical therapy, as well as shortened transport and PCI procedural times.

In the present study, we have shown that even in the most severe subgroup of ACS patients (CS and ACS), TTECG-facilitated triage had beneficial effects on the in-hospital survival rate. ECG changes in ACS are known to be highly dynamic. Therefore, the early acquisition and transmission of ECG data in ACS can provide useful and critical information to increase the accuracy of the diagnosis. With reference to recent guidelines, a 12-lead ECG is indispensable, and it must be interpreted as soon as possible at the time of the first medical contact of suspected ACS patients, to facilitate an early diagnosis (class IC recommendation), triage, and therapy [2,3]. To this end, TTECG, combined with teleconsultation, appears to be an optimal tool. It was previously shown that early pre-hospital triage with telemedicine in subjects with STEMI was linked to higher rates of timely reperfusion [20] and lower mortality rates in both observational studies [6,21,22] and meta-analyses [23,24]. These unexpected findings on mortality rates were suggested to be mediated by reduced ischaemic time and better pre-hospital care, resulting from TTECG-based triage [25]. Corresponding to these findings, the present study also confirms that TTECG-based teleconsultation and triage are independent predictors of lower in-hospital mortality rate, and this also applies for high-risk ACS patients with CS.

What could be the causal relationship between TTECG-based triage and improved survival in ACS patients with CS? Based on our current data, TTECG-based triage was associated with a drastically reduced time interval between symptom onset and hospital admission. Evidently, the earlier the reperfusion, the better the myocardial function (i.e., “time is muscle”). Moreover, other important time intervals (door-to-sheath insertion and door-to-balloon time) have also been found to be reduced by TTECG, as shown in both the present study and previous investigations [6]). Finally, yet another important benefit of TTECG-based triage could be that prehospital pharmacotherapy is discussed by the emergency personnel and the PCI centre staff via teleconsultation [6,19].

The hospital medical therapy in our study was administered according to current guidelines [10]. Each patient in the two groups received inotropes and/or vasoconstrictors. After the stabilization of blood pressure (>90/70 mmHg), levosimendan was initiated when necessary [26].

It is generally accepted that total occlusions and intracoronary thrombi are directly linked to lower coronary flow, myocardial damage, and loss of function. At the same time, improved coronary flow leads to better myocardial protection and function; consequently, to increased survival rate. Recent ESC Guidelines on myocardial revascularization [14] and acute and chronic heart failure [10] underline the significance of early and complete revascularization in order to improve perfusion. The QuBE score values have been shown to have a close correlation with perfusion and microvascular function [18]. Moreover, it was noted that the QuBE score provides a useful surrogate endpoint in trials of therapies that attempt to improve myocardial reperfusion [17]. Indeed, in accordance with the guidelines’ proposal, our current results indicate that better perfusion in the affected myocardium (high AAR QuBE score/AAR segment number) after PCI is an important determinant of the survival rate of ACS patients with CS.

Cardiogenic shock is initially characterized by a failure to maintain global oxygen delivery. However, the progression of CS is associated with derangement in the regulation of regional blood flow, microcirculatory abnormalities, and cellular hypoxia [27]. Early restoration of myocardial perfusion may limit the development of microcirculatory and cellular abnormalities. In accordance with this approach, multiple registries [28,29] and clinical studies [30] have confirmed the effectiveness (significant decrease in mortality) of early revascularization in CS. In fact, the SHOCK trial failed to meet the primary endpoint (lowering 30-day mortality) with early revascularization in comparison to initial medical treatment. However, there was a significant mortality reduction after 6 months, 1 year, and 6 years [31,32].

In the present study, the prolonged time interval between symptom onset and hospital admission, the successful resuscitation of ACS patients and high CK_max_ value were identified as useful independent predictors for in-hospital mortality rate. These findings correspond to the results demonstrated by previous reports [7,10].

## 5. Conclusions

The main goal of our present study was to determine the impact of various parameters (prehospital TTECG-based teleconsultation, LV function, coronary perfusion, and blood chemistry) on mortality in a retrospective analysis of patients suffering an ACS episode complicated with CS, treated with guideline-based complex therapy. Our research is the first to demonstrate that the TTECG-facilitated diagnosis and triage of patients is an important independent predictor of a higher in-hospital survival rate in this population.

Previously, several studies have shown that the prehospital telemedicine triage of ACS patients speeds up diagnosis while optimizing first-line medical therapy, especially if cardiologists are also involved in the prehospital teleconsultation [5,6]. Furthermore, we have shown that telemedicine facilitates the more efficient organization of patient transport and the preparation of hospital staff for emergent PCI, thereby reducing the time necessary for revascularization. Early coronary artery revascularization results in better LV function and CS outcomes, a finding also supported by the results of our current study. We also showed that improved perfusion of the affected myocardium after PCI is an important determinant of patient survival rates. Moreover, a high GFR value at the time of hospital admission was identified as an independent predictor of better survival, highlighting the importance of preserved function in other organs than the heart in favor of an improved clinical outcome.

To summarize, TTECG-based teleconsultation plays an indisputable role in the establishment of a timely ACS diagnosis and rapid transport of patients; hence, the prompt revascularization of the infarct-related artery and ultimately, the preservation of myocardial function and improved survival of patients with CS. Thus, in the future, it is encouraged to exploit the beneficial effects of telemedicine not only in ambulatory settings, but in the care of acute cardiac events as well.

## 6. Limitations

A potential limitation of this study is that all data analyses were performed on a retrospective basis and on a relatively small sample size (hospital death group; *n* = 77). It should be emphasized, however, that during data collection, a relatively long inclusion time (4 years) was applied and all ACS patients with CS were included in the database. The respective groups (hospital death group and survival group) were considerably well-matched regarding age, gender, risk factors, previous medical history, medical therapy, and stent procedural details.

As the data collection period was 2009–2012, new improvements may have appeared since then both in pharmacological and interventional therapies (new drug-eluting stents or drugs) that were not taken into consideration in our study. Furthermore, other relevant variables may not have been incorporated into our model either.

## Figures and Tables

**Table 1:** Clinical characteristics of acute coronary syndrome patients with acute heart failure.

	Hospital Death Group (*n* = 77)	Hospital Survival Group (*n* = 210)	*p* Value
**General**			
Age (yrs)	66.04 ± 12.56	63.60 ± 11.98	0.1425
Men (%)	59.74	61.90	0.7318
TTECG-based consultation (%)	27.27	45.71	0.0104
Time interval between symptom onset and hospital admission (h)	26.63 ± 54.93	13.16 ± 25.35	<0.0001
Door-to-balloon time (min) ^+^	68.24 ± 39.03	60.06 ± 29.25	0.1369
Resuscitation (%)	24.67	14.28	0.1775
LVEF (%)	30.28 ± 8.37	36.96 ± 8.67	<0.0001
AAR (%)	66.62 ± 24.59	61.95 ± 24.67	0.1571
LV QuBE score (arbitrary units) ^§^	115.50 ± 33.31	160.13 ± 38.70	<0.0001
AAR QuBE score (arbitrary units) ^§^	55.95 ± 30.16	90.11 ± 46.08	<0.0001
AAR QuBE score/AAR segment number (arbitrary units) ^§^	4.97 ± 2.28	8.89 ± 3.43	<0.0001
**Blood Chemistry**			
GFR (mL/min)	52.71 ± 23.39	66.25 ± 22.33	<0.0001
CK_max_ (U/L)	5141.27 ± 8247.84	2166.47 ± 2607.36	0.0038
Haemoglobin (g/L)	130.17 ± 21.18	135.56 ± 19.82	0.0625
Thrombocyte number ×10^3^ (µL)	241.50 ± 72.90	259.10 ± 72.70	0.2459
**Previous Cardiac Risk Factors (%)**			
Smoking	29.87	31.42	0.8178
Hypertension	64.93	68.09	0.6052
Diabetes mellitus	35.06	32.38	0.7616
PAD	19.48	25.71	0.3931
**Proportion of Patients (%) with a Previous History of**			
Myocardial infarction	23.37	28.57	0.5002
Congestive heart failure	16.88	20.00	0.7318
PCI	10.38	9.52	0.9105
Coronary artery bypass graft surgery	3.89	3.33	0.7961

Values are means ± SD or percentages of subjects. AAR = area at risk; GFR = glomerular filtration rate; LV = left ventricle; LVEF = left ventricular ejection fraction; PAD = peripheral artery disease; PCI = percutaneous coronary intervention; QuBE = myocardial blush grade; TTECG = transtelephonic ECG. In this instance, the *p* value refers to differences between the hospital death group and the hospital survival group. ^+^ The door to balloon time was available only if the PCI has been performed (*n* = 64 and 155 in the hospital death group and hospital survival group, respectively). ^§^ In case of QuBE calculations the number of patients (*n*) were 66 and 156 in the hospital death group and hospital survival group, respectively.

**Table 2:** Stent procedural details and medical therapy in the acute phase (first week).

	Hospital Death Group (*n* = 77)	Hospital Survival Group (*n* = 210)	*p* Value
**Stent procedural details**			
Proportion of STEMI patients (%)	83.12	71.43	0.1293
One-vessel disease (%)	64.93	54.29	0.1069
Proportion of stented patients (%)	83.12	73.81	0.1010
Stent/patient (mean ± SD)	1.24 ± 0.50	1.28 ± 0.53	0.2945
Drug-eluting stent (%)	5.19	10.00	0.1010
*LM (%)	3.13	3.23	0.9693
*LAD (%)	68.75	76.77	0.2168
*CX (%)	18.75	23.87	0.4093
*RCA (%)	29.68	21.29	0.1851
**Medical Therapy in the Acute Phase (%)**			
Inotropes (dobutamine)	97.40	94.76	0.7318
Vasopressor (norepinephrine/dopamine)	100.00	100.00	1.0000
Levosimendan	9.19	10.00	0.9597
GP IIb/IIIa inhibitor	51.95	44.76	0.2708

Values are in mean ± SD or percentages of subjects. CX = left circumflex coronary artery; GP IIb/IIIa inhibitor = glycoprotein IIb/IIIa inhibitor; LAD = left anterior descending coronary artery; LM = left main stem; RCA = right coronary artery; STEMI = ST-segment elevation myocardial infarction. * Patients may have had interventions on more than one vessel.

## Data Availability

The data presented in this study are available on request from the corresponding author. The data are not publicly available due to privacy.
